# System Interface for an Integrated Intelligent Safety System (ISS) for Vehicle Applications

**DOI:** 10.3390/s100201141

**Published:** 2010-01-29

**Authors:** Mahammad A. Hannan, Aini Hussain, Salina A. Samad

**Affiliations:** Department of Electrical, Electronic & Systems Engineering, National University of Malaysia, 43600 Bangi, Selangor, Malaysia; E-Mails: aini@eng.ukm.my (A.H.); salina@eng.ukm.my (S.A.S.)

**Keywords:** system interface, intelligent safety system, ADDS, TPMS, integration

## Abstract

This paper deals with the interface-relevant activity of a vehicle integrated intelligent safety system (ISS) that includes an airbag deployment decision system (ADDS) and a tire pressure monitoring system (TPMS). A program is developed in LabWindows/CVI, using C for prototype implementation. The prototype is primarily concerned with the interconnection between hardware objects such as a load cell, web camera, accelerometer, TPM tire module and receiver module, DAQ card, CPU card and a touch screen. Several safety subsystems, including image processing, weight sensing and crash detection systems, are integrated, and their outputs are combined to yield intelligent decisions regarding airbag deployment. The integrated safety system also monitors tire pressure and temperature. Testing and experimentation with this ISS suggests that the system is unique, robust, intelligent, and appropriate for in-vehicle applications.

## 1. Introduction

In any vehicle, the presence of intelligent safety implies an active system that promotes safety, security and driving comfort [1]. However, to meet high expectations for control and safety, a large number of individual safety systems are required [2,3]. This has led to concern over safety issues and has resulted in a need for integrated ISSs that feature effective new technologies, characterize safety issues and provide solutions for monitoring, detecting, and classifying impending crashes or unsafe driving conditions. The ISS should warn the driver, improving his or her ability to control the vehicle and thereby preventing accidents [4,5].

In the past, many researchers have adopted approaches towards individual safety issues such as the detection, classification and location of occupants, vehicle crash detection and severity analysis, TPMS, *etc.* For example, in [3,6,7] occupant detection and characterization parameters were studied to improve the safety and comfort features for all occupants. However, the challenge of detecting and distinguishing a particular class of occupant from all others remains daunting. Despite the success of some of these systems, occupant detection and classification involving human subjects and non-human objects still poses a number of challenges, and further progress remains necessary for addressing changes in illumination, image scale, image quality, expression and pose. Sensors for data acquisition, real time implementations, and operations should also be studied further [8].

Crash detection is a helpful concept in preventative safety, preventing accidents, collisions and minimizing human injury when an accident occurs [5,9]. In the past, practical crash detection has not been widely discussed, and researchers have mainly considered the theoretical aspects of crash analysis using traditional engineering principles [10–12]. Recently, several attempts have been made to develop an automated system to detect vehicle crashes, vehicle types and crashes under various conditions such as during and after heavy downpours, driving at dawn or at dusk, sunlight reflections, vehicles driven at high speeds and out of position. These are considered as high risk problems that require dedicated solutions. Before now, automated solutions were not feasible or did not perform sufficiently robustly for everyday use [12]. If these problems are not addressed properly, they will continue to serve as obstacles to the implementation of intelligent crash detection systems. Therefore, the national highway traffic safety and administration (NHTSA) and other road related safety authorities have called for the mandatory consideration of crash detection and analysis as a key safety issue [13,14].

Similarly, TPMS performance is important for improving both driving experience and vehicle performance [15]. Vehicles without TPMS features have more safety problems. To date, a number of TPMS have been widely investigated in order to solve the problems. Major concerns include limited lithium battery lifetimes, malfunctioning of the electromagnetic RF transceiver unit, echo-based noise due to broadcasting pulse responses, inadequate sensor capabilities, and low robustness in harsh environments encountered during vehicle operation [16,17]. In particular, appropriate sensors for different TPMS applications are still under investigation [18,19]. Accordingly, in the TREAD act the NHTSA legislated that, after 31 October 2006, all vehicles in the United States must offer TPMS as an option [13,20–22].

The fields of intelligent vehicles and their applications are rapidly growing worldwide, as is interest from the automobile, truck, public transportation, industrial, and military sectors. The ISS offers the potential to significantly enhance both safety and operational efficiency [23,24]. Increasing demand for quality ISS solutions has driven the design of robust safety technologies, the study of safety issues and the provision of solutions that involve monitoring, detecting, and classifying impending crashes or unsafe driving conditions, and by warning the driver, improving his or her ability to control the vehicle and prevent an accident [3]. In intelligent transportation systems, ISSs use sensing and intelligent algorithms to understand the vehicle’s immediate environment, either assisting the driver or fully controlling the vehicle. However, state of the art studies of prototype integrated ISSs suggest that there remains a gap between many of these inventions and actual marketable products [25,26]. For such products or inventions to be effective, we believe that a robust system is required for interfacing a given ISS prototype implementation with other vehicle components. Therefore, in this paper, we highlight the importance of good system interfaces, and demonstrate their use in the development of an innovative integrated ISS. This ISS can identify major hazards and can assess the associated risks in various environments where more traditional tools cannot be effectively or efficiently applied. Safety devices provide data to the ISS that are useful for the development of ADDS and TPMS. This paper successfully integrates and develops an advanced ISS with such features as occupant detection, classification and positioning, vehicle crash detection, crash severity analysis, tire pressure monitoring, and analysis of other hazards.

## 2. System Integration

The main motivation behind system integration is to reduce the management costs of individual safety systems, which translates into improved system performance. Further, system integration reduces the programming resources necessary to meet response time requirements and to maintain a high service quality. Performance tuning is accomplished by obtaining information about how much time is spent on each safety measures of a distributed transaction, as well as information about the delays that might occur in the overall integration process. The integrated ISS aims to provide heterogeneous workload management concepts and functions to addresses safety issues based on diagnoses in a developed platform using collected monitoring data. The hardware platform identifies a set of hardware objects, each associated with a processor. The system interface provides a high level of interfacing between software running on different processors that control the hardware. The major tasks of the integrated ISS include performance characterization, problem determination and real workload data monitoring of distributed safety issues that are incorporated into the system. The proposed ISS deals with safety and comfort concerns in the modern vehicle, including tire pressure monitoring, occupant detection, crash detection and vehicle position monitoring. This integrated ISS gathers environmental data using a set of sensors, collected the data through acquisition processes, eventually reacts through a CPU, and finally outputs information on safety issues to a LCD display unit.

## 3. Algorithm and Methodology

Methods and algorithms for the ISS were developed for ADDS and TPMS, which involved the individual algorithms for occupant detection, classification and position based on weight sensing and image processing as well as for vehicle crash detection. For classification purposes, weight measurement data are used with additional logic elements. For example, when an adult occupant is on a seat, the adult logical variable is set to true, child and non-human object logical variables are set to false, the algorithm classifies the occupant as an adult and displays relevant output data on the monitor. For position detection, we calculated the centroidal distances of *Fx* and *Fy* as follows [27]:

(1) $$Fx=x\frac{\left(-F1+F2-F3+F4\right)}{\left(F1+F2+F3+F4\right)}$$

(2) $$Fy=y\frac{\left(F1+F2-F3-F4\right)}{\left(F1+F2+F3+F4\right)}$$

where *F*1, *F*2, *F*3 and *F*4 are weights as detected by the four weight sensors, while x and y indicate the distances from the centre to the sensor in the *x* and *y* directions, respectively. The output of the calculations involving *Fx* and *Fy* gives the position of the occupant.

**Figure 1.**
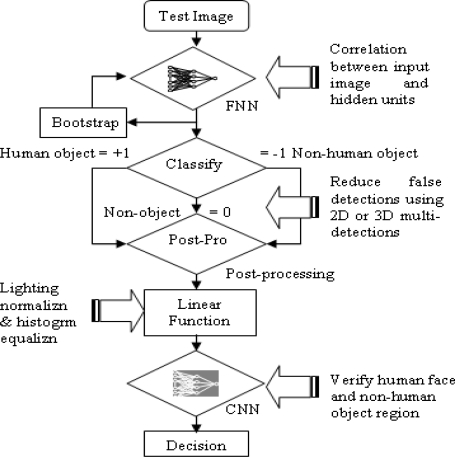
Neural network algorithm for occupancy detection.

The algorithmic approach based on image processing for the detection and classification of occupant, non-human object and non-object is shown in Figure 1.

The proposed system is a combination of a fast neural network (FNN) and a classical neural network (CNN). The FNN analyzes any image for which a positive detection has been made, including false positive identifications. CNN is used to verify the region of detection. Under the proposed system architecture, the FNN extracts a sub-image from the test image to distinguish between correct object and false detections. Post-processing strategies are applied to convert normalized outputs back into consistent units and to eliminate detection overlap. Initially, we assumed that the FNN could be confounded into false detection by variable lighting conditions. For example, illuminating the side of an object changes its overall appearance. To solve this problem, an automatic linear function was initially used to adjust image intensity values using histogram equalization or lighting corrections. However, neither method was found to be suitable. Rather, an alternative method was used that employed an object verification procedure using the CNN. This CNN object verifier helped reduce false detection rates. This combined network was capable of higher detection accuracy and exhibited better computational efficiency compared to a single network, which was unable to fully eliminate the false detection problem.

The change in vehicle velocity, Δ*v*(*t*), is an essential parameter for crash detection and is used here in the development of our crash detection algorithm. Δ*v*(*t*) is obtained by integrating the acceleration signal [28] as shown below.

(3) $$\Delta v(t)=\int a\;(t)\;dt=A\;\omega^{2}\int \text{cos}\;\;\;(\omega \;t+\delta )$$

A suitable vehicle velocity threshold, *V_th_*, is required to facilitate decision making as to whether or not a crash has effectively occurred. This threshold value *V_th_* can easily be determined from the lowest effective speed of a crash as defined by NHTSA, which is 22.54 km/h. To detect a crash, the following algorithmic steps were used:

- If Δ*v*(*t*) ≥ *V_th_*, then output = ‘1’; DECISION: Effective crash is detected.
- If Δ*v*(*t*) < *V_th_*, then output = ‘0’; DECISION: Effective crash is not detected.

The change of velocity Δ*v*(*t*) over a period of time T can easily be computed for this decision since the integral over the noise component is approximately zero. The circuit for computing Δ*v*(*t*) can be designed using systolic architecture to determine the real-time speed. The systolic design processes the output data in the systolic array for required operation of the optimal detection state. The detection state is fed into a data acquisition card for system development.

For the TPMS, a threshold check algorithm is used to acquire data from the sensors. For the threshold check, the DAR is preloaded with a threshold value while in standby/reset mode to detect whether the pressure or temperature has crossed a particular level. The receiver module is capable of receiving both on-off keying (OOK) and frequency shift keying (FSK) inputs through a UHF receiver that communicates with the CPU via an SPI. The UHF receiver detects and demodulates the signal through a Manchester-encoded bit stream, sending the important data out to the CPU. Data is then monitored in the display unit. The TPM and receiver modules are loaded with a simple software program to improve the functionality of the hardware. The assembly code for the TPM module is written using the “WIN IDE” integrated development environment and is programmed into RF2 using a programmer board that transmits data to the receiver module. The receiver module communicates with the UHF receiver using a Turbo C compiler under DOS. The “TPMReceiverModule” function is created in the main interface program UKM.dll to monitors pressure and temperature data transmitted from the TPM receiver through the SPI connection to the CPU.

## 4. Prototype Structure

**Figure 2.**
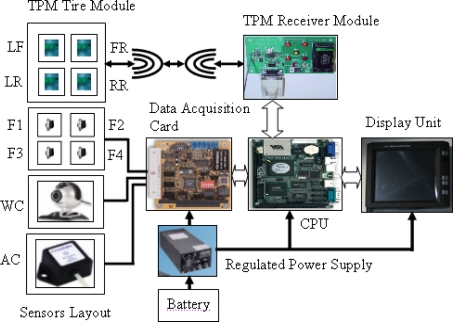
Integrated prototype system hardware.

The hardware prototype is a complete representation of the final design of the integrated ISS, simulating its real-time behavior. This system implementation was developed by making physical interconnections between hardware objects using standard hardware design techniques. The system consists of the following hardware objects: sensors, tire pressure monitoring modules, a load cell weight sensor, a Logitech web camera, a Cross-bow accelerometer crash sensor, a data acquisition card for analog to digital conversion, a CPU card, a touch screen for deploying results and an ATX switched mode power supply (SMPS) as shown in Figure 2.

## 5. System Interface Program

**Figure 3.**
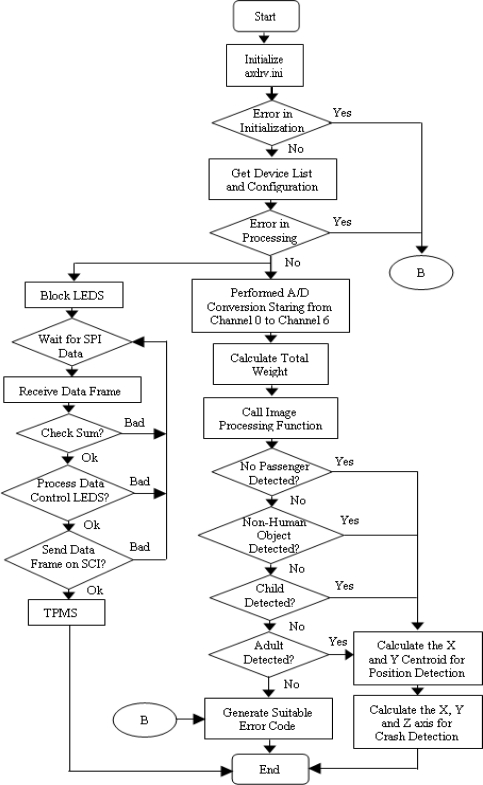
Program flowchart for the integrated system.

In the interface program, data were acquired from the weight sensor inside the passenger seat and from the crash accelerometer fixed on the vehicle bumper through an AXIOMTEK AX10410A acquisition card. The weight and crash sensors provided analog signals that were received by CH0 through CH6 of the A/D converter on the DAQ card. A web camera was connected to the CPU via a USB interface. The system interface between the software and hardware was developed in C using the LabWindows/CVI software. The low level driver “c:\cvinterface\UKM.dll” was written as a Win32 DLL file such that the functions inside the DLL were called by the Lab Window/CVI C code. In this DLL file, the *“Func1”* function processed the analog signals received by CH0 through CH6 of the A/D converter on the DAQ card. The *“HumanDetection”* function decided, based on weight sensing, whether the seat was occupied and, if so, whether by an adult or a child. The function *“ImageProcess”* was called inside UKM.dll to detect a person. This function returned a 1 if the image captured by the web-cam was determined to be “human.” If it detected a *“*non-human object*”* the function returned −1, and it returned 0 if it detected no object. The resulting 1, 0 and −1 values were fused with the logic combination of the weight sensor to determine the identity of the occupant—whether adult, child, non-human object, or nonexistent. The function *“CrashSensor”* was responsible for determining whether a crash occurred. The *“PositionDetection”* function calculated the centroidal distance of the object from the x and y axes, worked with UKM.dll to display a GUI, and made decisions regarding occupant position. Finally, the function *“ABagParm”* provides the airbag deployment decision upon fusing logic combination of occupant classification, position and vehicle crash detection decision. The function *“TPMReceiverModule”* called in UKM.dll monitored pressure and temperature, as extracted from the TPM receiver through the SPI connection to the CPU. Figure 3 shows a detailed program flowchart diagram for the UKM.dll.

## 6. Results and Discussion

To assess the performance of the ISS, we evaluated its network interface processing, its image and signal processing for the purpose of occupant detection, its classification and positioning, its vehicle crash detection accuracy, its severity analyses for ADDS and its TPMS performance monitoring. Typically, real-time constraints can be as large as 1 minute. However, in our prototyped hardware, the execution vectors for the whole system were derived from experimental measurements within 50 ms.

We used two sets of experimental image data to assess ISS detection performance between human and non-human objects. These images were distinct from the training sets. Human detection was performed based on human face detection. The first set consisted of 253 test images of human faces against complex backgrounds, variously scaled and with variations in lighting. The second data set contained 112 non-human object test images. The system underwent a bootstrapping cycle, to evaluate the true performance of the detection algorithm and the rate of false detections from images of natural scenes that did not contain human faces or non-human object.

Table 1 shows the performance of various human detection algorithms using test set 1. Our results are compared with other systems over a variety of metrics, including the number of faces detected, faces missed, faces falsely detected and computation time. The success rate of the proposed method was 97.6%, with six false alarms. We note that the number of false alarms was quite small compared with Ben-Yacoub *et al.* and Fasel *et al.* methods [29,30]. The improved performance of Rowley *et al.* [31] is likely due to the size of the training data, but this technique is less efficient than ours in term of false detection. On the other hand, Yacoub *et al.* demonstrated an algorithm with a very fast processing time but also with a high incidence of false alarms as well as a lower detection rate [29].

Table 2 summarizes detection results from the non-human object test set 2, compared with other systems. Our algorithm successfully detected 96.42% of non-human objects, with 3.58 false alarms. This value is lower than those obtained by Agarwal *et al*., Mahmud and Herbert and Viola and Jones [32–34]. Based on the results shown in Tables 1 and 2, we conclude that our algorithm makes acceptable tradeoffs between the number of false detections and the processing time, both for humans and for non-human objects.

After completing the image processing task, the *“ImageProcess”* function returned a value of 1 indicating a human, −1 indicating a non-human object and 0 indicating a non-object. The integrated ISS made the seat occupancy decision based on the assigned value. If the seat was deemed occupied, the system performed occupant classification, and classified the occupant as an adult, child or non-human object. Typically, human occupants generate weight information that varies in position as a function of time. However, non-human objects like grocery bags are static and yield weight information without positional variation. Using this information, the occupant’s position can be determined and used to measure comfort level and to assess risks *versus* benefits of airbag deployment in the event of a crash.

**Figure 4.**
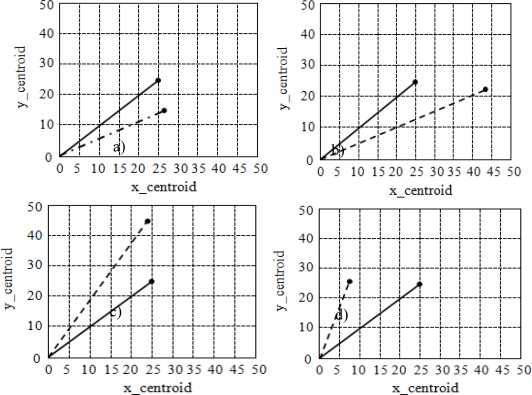
Occupant centroidal position calculations.

Figure 4 shows various centroidal positions of the vehicle’s front passenger using values of *y_centroid vs. x_centroid* for a seat size of 50 × 50 cm. *y_centroid* represents the forward direction while *x_centroid* represents left-right movement. The solid line indicates the ideal position whereas the dashed line represents the actual occupant position. If the endpoint of the dashed line lay within a radius of 10 cm from the centroid (at coordinates 25, 25), then the occupant position was categorized as good. Any centroid outside this radius was classified as a bad position. Figure 4a represents a well-positioned occupant, while Figure 4b,c,d are classified as out of position and are regarded as bad. These results can be further interpreted. For instance, from Figure 4a we conclude that the occupant is seated in a good position and that his or her back is properly positioned and aligned with respect to the seat. Figure 4b illustrates that the occupant is positioned very much to the right of the seat. On the other hand, Figure 4c illustrates that the occupant is leaning forward and is in close proximity to the airbag. In this case, the ISS would choose not to deploy the airbag since it is capable of doing significant harm to the occupant. Similarly, Figure 4d implies that occupant is seated far to the left in the seat.

**Figure 5.**
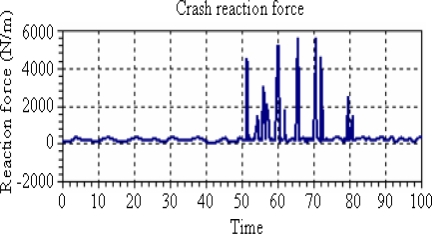
Vehicle crash reaction forces.

Finally, crash severity analysis is investigated. Experimental results of crash reaction forces are shown in Figure 5. It is seen that the repeated crashes occurred between 51 sec and 80 sec, and reaction forces obtained during this time interval were about ∼1,000 N/m to ∼5,800 N/m. These values are greater than the threshold value of 22.54 km/h. Since reaction force depends on crash velocity, a higher velocity implies an increase in reaction force, which in turn increases crash severity. This then puts the occupant at higher risk.

**Figure 6.**
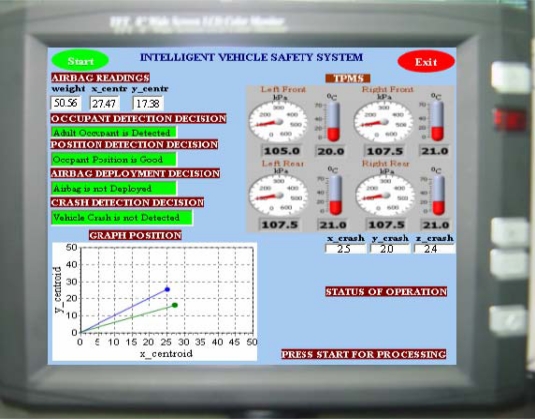
Display interface for the integrated ISS.

Figure 6 shows the display outputs for the experimental results of the implemented integrated ISS for ADDS and for TPMS. The safety feature functions are activated by pressing the start button. If the system does not detect a crash, it will ignore the occupant detection results. Accordingly, if a crash is detected and classified as severe, airbags will deploy accordingly based on the results of the occupant detection module.

If this module detects a non-human object, the airbag will not be deployed, and if it detects a human object, further classification will be made to determine whether or not the occupant is an adult. All decisions are highlighted in green on the display unit. For the TPMS, the display unit shows acquired real-time temperature and pressure data. Thus, the integrated prototype consistently calculates the optimum fused decision based on a broad assessment of risk, a function that is very useful for a vehicle assistance system.

## 7. Conclusions

This study integrates various theories and methodologies implemented in vehicle safety systems into a unique, original platform. Ultimate goal was the complete integration of a prototypical vehicular ISS, including a TPMS, which in turn is able to promote the safety, security and comfort of vehicular occupants. The interfaces of the integrated prototype were presented. A Lab Window/CVI interface program coded in C was used to implement this real-time intelligent safety system prototype. The safety components such as occupant detection, classification and location, vehicle crash detection and TPMS were integrated. Algorithms and methodologies were developed for the hardware platform and the system interface program. Use of the embedded ISS resulted in a successful real-time working device, which provides intelligent safety management and functional performance. The proposed prototype offers advantages in terms of performance characterization, problem determination and real-time data monitoring, as well as in providing vehicle safety warnings.

## Figures and Tables

**Table 1.** Detection rate for set 1, using different methods.

**Method**	**Detected Humans (%)**	**Missed Humans (%)**	**No. of False Detections**	**Processing Time**
FNN+CNN	97.63%	2.37%	6	2.3 s
Rowley *et al.*	97.86%	2.14%	13	0.013M
Yacoub *et al.*	84.31%	15.69%	347	0.7 s
Fasel *et al.*	96.8%	3.2%	278	3.1 s

**Table 2.** Detection rates for set 2, using different methods.

**Method**	**Non-Human Object Detections**	**Missed Non-Human Objects**	**No. of False Detections**	**Processing Time**
FNN+CNN	96.42%	3.58%	4	2.9 s
Agarwal *et al.*	94%	6%	30	3.6 s
Mahmud and Hebert	82%	18%	187	4.0 s
Viola and Jones	95%	5%	71	0.7 s
